# Dynamic state allocation for MEG source reconstruction

**DOI:** 10.1016/j.neuroimage.2013.03.036

**Published:** 2013-08-15

**Authors:** Mark W. Woolrich, Adam Baker, Henry Luckhoo, Hamid Mohseni, Gareth Barnes, Matthew Brookes, Iead Rezek

**Affiliations:** a Oxford Centre for Human Brain Activity (OHBA), University of Oxford, Warneford Hospital, Oxford, UK; b Centre for Functional MRI of the Brain (FMRIB), University of Oxford, John Radcliffe Hospital, Oxford, UK; c Wellcome Trust Centre for Neuroimaging, UCL, London, UK; d Sir Peter Mansfield Magnetic Resonance Centre, School of Physics and Astronomy, University of Nottingham, University Park, Nottingham, UK; e Department of Engineering Science, University of Oxford, Parks Road, Oxford, UK

**Keywords:** Magnetoencephalography, MEG, EEG, Source reconstruction, Microstates, Connectivity, Hidden Markov Model

## Abstract

Our understanding of the dynamics of neuronal activity in the human brain remains limited, due in part to a lack of adequate methods for reconstructing neuronal activity from noninvasive electrophysiological data. Here, we present a novel adaptive time-varying approach to source reconstruction that can be applied to magnetoencephalography (MEG) and electroencephalography (EEG) data. The method is underpinned by a Hidden Markov Model (HMM), which infers the points in time when particular states re-occur in the sensor space data. HMM inference finds short-lived states on the scale of 100 ms. Intriguingly, this is on the same timescale as EEG microstates. The resulting state time courses can be used to intelligently pool data over these distinct and short-lived periods in time. This is used to compute time-varying data covariance matrices for use in beamforming, resulting in a source reconstruction approach that can tune its spatial filtering properties to those required at different points in time. Proof of principle is demonstrated with simulated data, and we demonstrate improvements when the method is applied to MEG.

## Highlights

• Novel adaptive time-varying approach to MEG source reconstruction
• HMM infers reoccurring short-lived states on the same scale as EEG microstates
• Tunes source reconstruction properties to those needed at different points in time
• Improves spatial localisation of high temporal resolution information from MEG data

## Introduction

Magnetoencephalography (MEG) and electroencephalography (EEG) data have the ability to provide direct, non-invasive measurements of neuronal activity. This is providing new insights into the dynamics of brain activity at the systems level, most recently using magnetoencephalography (MEG) to investigate networks of oscillatory activity in the human brain (Brookes et al., 2011, de Pasquale et al., 2010, Hipp et al., 2012, Luckhoo et al., 2012). However, while the temporal information is excellent, in many circumstances the spatial resolution is relatively poor.

Beamforming is a commonly used method for performing source reconstruction of brain activity, particularly for oscillatory activity. Existing implementations typically correspond to a temporally stationary, spatially adaptive spatial filter (Van Veen et al., 1997). The beamformer spatial filter weights are determined from the forward model, i.e. the lead field matrix, and an estimate of the sensor data covariance. The accuracy of this data covariance matrix estimation is therefore crucial, and key to the beamformer's ability to spatially adapt to the data (Brookes et al., 2008).

The data covariance matrix is estimated typically by pooling all of the available data over time, which implicitly assumes that the data is temporally stationary. However, this assumption is at odds with what we know about MEG and EEG data. Firstly, there are likely to be temporal non-stationarities in artefacts, e.g. the variation over time in physiological activity such as the cardiac cycle. Secondly, there is increasingly strong evidence of temporal non-stationarity in neuronal activity, even in the resting state, from both fMRI (Smith et al., 2012) and MEG (de Pasquale et al., 2010) data. In EEG, there is evidence of scalp topographies that remain quasi-stable for periods of about 100 ms, known as EEG microstates (Britz et al., 2010, Koenig et al., 2005, Van de Ville et al., 2010).

Techniques such as Independent Component Analysis (ICA) can be used to remove temporally non-stationary artefacts, e.g. due to cardiac and respiratory cycles, in sensor space prior to source reconstruction (Mantini et al., 2011). However, this still potentially leaves time-varying neuronal phenomenon unaccounted for. Motivated by the need to adapt to general temporal non-stationarities inherent in electrophysiological data, Dalal et al. (2008) proposed using data covariance matrices tuned to specific time (and frequency) windows. However, this introduces a trade-off between the need for large integration windows to give good data covariance estimation (Brookes et al., 2008), and the desire to focus on a specific time window. While there are approaches that can adaptively tune the regularisation of the data covariance matrix estimation in these settings to ensure stable performance (Wipf and Nagarajan, 2007, Woolrich et al., 2011, Zumer et al., 2007), the trade-off still remains.

In this paper we propose a novel adaptive time-varying approach to data covariance estimation, designed to handle temporally non-stationary electrophysiological data. This uses a Hidden Markov Model (HMM) to infer when in time particular states re-occur in the sensor space data. The resulting HMM state time courses can then be used to intelligently pool data over distinct and potentially short-lived periods in time, to compute time-varying data covariance matrices. These can then be used to compute time-varying spatial filter beamformer weights tuned to each point in time.

## Methods

### Hidden Markov Model

HMMs model data as being generated from any one of a number of discrete states. The states are “hidden”, i.e. not directly observable. However, associated with each state is an observation model that consists of a probabilistic mapping of each state to the observed data. In this work, the hidden states may correspond to periods of time when a particular artefact, or a potentially different neuronal brain state, is present.

We assume a HMM of length *T*, state space dimension *K*, hidden state variables, *s* = {*s*_1_ … *s*_*T*_}, and M/EEG sensor data, *y* = {*y*_1_ … *y*_*T*_}, where *y_t_* is the *N* × 1 M/EEG sensor data at time *t* with *N* equal to the number of M/EEG sensors. The full true posterior probability of the model is then given by

(1) $$P\left(y,s,\theta ,\pi \right)=P\left(s_{0}|\pi_{0}\right)\prod_{t}P\left(s_{t}|s_{t-1},\pi \right)P\left(y_{t}|s_{t},\theta \right)P\left(\theta \right)P\left(\pi \right)$$

where *P*(*θ*), and *P*(*π*) are chosen to be non-informative priors, and *π*_0_ is the initial state probability. We have assumed that the probability to transition to another state depends only on the state that the system is in, and not on the path it took to get to its current state, i.e. it is Markovian:

(2) $$P\left(s_{t}|s_{1}\ldots s_{t-1}\right)=P\left(s_{t}|s_{t-1}\right)=\pi$$

where *π* is the *K* × *K* transition probability matrix, in which the element (*k*, *j*) describes the probability of transitioning from state *k* to state *j* between time *t* − 1 and time *t*.

The term, *P*(*y_t_*|*st*, *θ*), is the observation model that describes the distribution of the data for each of the states *s_t_*. In this work we assume that the observation model for state *k* is a multivariate Normal distribution (MVN) with *θ_k_* = {*μ_k_*, Σ*_k_*}, where *μ_k_* is the (K × 1) mean vector, and Σ*_k_* is the (K × K) covariance matrix:

(3) $$P\left(y_{t}|s_{t}=k,\theta \right)\sim \mathrm{MVN}\left(\mu_{k},\Sigma_{k}\right)$$

where *MVN* is a multivariate Normal distribution. A Hidden Markov Model can be considered a generalisation of a mixture model where the state variables are related through a Markov process rather than being independent of each other. Indeed, a Hidden Markov Model in space rather than time, corresponds to a Markov Random Field as used in spatial mixture modelling (Woolrich and Behrens, 2006, Woolrich et al., 2005).

#### Priors

The prior distributions over the HMM parameters, Θ = {*π*_0_, *π*, *θ*}, are chosen to be conjugate distributions. The approximate posterior distributions will then be functionally identical to the prior distributions (i.e. a Gaussian prior density is mapped to a Gaussian posterior density), making the model tractable to certain kinds of inference. See Rezek and Roberts (2005) for details.

#### Inference

In this work we use Variational Bayes (VB) inference on the HMM, as described in Rezek and Roberts (2005). This is fully probabilistic and furnishes us with the full posterior distributions of the model parameters, *P*(Θ, *s*|*y*). For the purpose of using the HMM inference to perform temporally adaptive beamforming, we could make use of the full probabilistic inference on *s_t_*, by using *P*(*s_t_*|*y*) as weighted averages in the temporally adaptive beamforming.

However, for computational efficiency we instead choose the most probable *a posterior* state, *u_t_*, at each time point:

(4) $$u_{t}=\underset{k}{\arg\max}P\left(s_{t}=k|y\right)$$

where *u_t_* is obtained using Viterbi decoding (which corresponds to the Maximum a posterior) (Rezek and Roberts, 2005). The state time course, *u_t_*, is then used to determine the pooling of data for computing the covariance matrices in the temporally adaptive HMM beamforming, as described in the next section.

##### Summary statistics

In assessing the output of the HMM inference it is useful to define some summary statistics. We have used the following measures.

*Fractional occupancy* is defined as the fraction of time spent in each state:

(5) $$\mathrm{Fractional}\;\mathrm{occupancy}\;\left(k\right)=\frac{1}{T}\sum_{t}\left(u_{t}==k\right)$$

where *u_t_* == *k* is one if *u_t_* = *k*, and is zero otherwise. The *Mean life-time* is defined as the amount of time spent in each state before transitioning out of that state:

(6) $$\mathrm{Mean}\;\mathrm{life}\;\mathrm{time}\;\left(k\right)=\frac{\sum_{t}\left(u_{t}==k\right)}{\mathrm{Number}\;\mathrm{of}\;\mathrm{Occurences}}$$

where the *Number of Occurrences* is given by:

(7) $$\mathrm{Number}\;\mathrm{of}\;\mathrm{Occurrences}\;\left(k\right)=\sum_{t}\left(\left(\left(u_{t}==k\right)-\left(u_{t-1}==k\right)\right)==1\right).$$

To aid in our evaluation of the method, we also use the *Symmetrised Kullback–Leibler (KL) divergence* to provide a measure of dis-similarity between the covariance matrices for the different states *k*, *j*:

(8) $$S_{\mathrm{KL}}\left(k,j\right)=0.5\left(D_{\mathrm{KL}}\left(f_{N}\left(0,\Sigma_{k}\right)\left\|f_{N}\left(0,\Sigma_{j}\right)\right)\right)+D_{\mathrm{KL}}\left(f_{N}\left(0,\Sigma_{j}\right)\left\|f_{N}\left(0,\Sigma_{k}\right)\right)\right)\right)$$

where *f_N_*(0, Σ*_k_*) is the pdf for a multivariate Normal distribution with zero mean and covariance matrix Σ*_k_*. This can be simplified to:

(9) $$S_{\mathrm{KL}}\left(k,j\right)=0.5\mathrm{tr}\left(\Sigma_{j}^{-1}\Sigma_{k}\right)+0.5\mathrm{tr}\left(\Sigma_{k}^{-1}\Sigma_{j}\right)-2N$$

where larger values of S_KL_(*k*, *j*) indicate larger differences in the covariance matrices.

### LCMV beamforming

We assume that the data points for which the *N* × *T* matrix of MEG signals, *y*, recorded at the *N* MEG sensors over *T* time points is modelled as

(10) $$y=\sum_{i}^{L}H\left(r_{i}\right)m\left(r_{i}\right)+e$$

where *H*(*r_i_*) is the *N* × 1 lead field matrix and *m*(*r_i_*) is the 1 × *T* vector time course for a dipole at location *r_i_*, *i* = 1 … *L*, and *e* ~ *N*(0, *C_e_*) is the noise with covariance which is assumed to be *C_e_* = *σ_e_*^2^*I*. Given this forward model we can use a beamformer to optimise the 1 × *N* spatial filter, *W*(*r_i_*), that estimates the dipole time course at location *r_i_* from the sensor data as

(11) $$\hat{m}\left(r_{i},t\right)=W\left(r_{i}\right)y\left(t\right).$$

A linearly constrained minimum variance (LCMV) beamformer has unit response in the pass band and minimises the variance in the stop band, resulting in the following solution for the weights matrix (Van Veen et al., 1997)

(12) $$W\left(r_{i}\right)={\left(H{\left(r_{i}\right)}^{T}C_{y}^{-1}H\left(r_{i}\right)\right)}^{-1}H{\left(r_{i}\right)}^{T}C_{y}^{-1}$$

This equation can be used at multiple locations to produce a whole brain image of the brain's activity. However, the sensitivity of the beamformer will vary for different locations within the brain. For example, deep sources will have much less sensitivity than superficial sources. The standard deviation of the estimator in Eq. (11) is calculated as the expected value of *m*(*r_i_*, *t*) *m*(*r_i_*, *t*)*^T^*, giving:

(13) $$\begin{array}{c} \mathrm{std}\left(\hat{m}\left(r_{i},t\right)\right)={\left(\hat{m}\left(r_{i}\right)\hat{m}{\left(r_{i}\right)}^{T}\right)}^{1/2}\\ ={\left(W\left(r_{i}\right)C_{y}W{\left(r_{i}\right)}^{T}\right)}^{1/2} \end{array}$$

Note that (for simplicity) the equations in this subsection correspond to a scalar beamformer with known dipole orientation. In practice, we determine the dipole orientation at each location by finding the projection of the lead field that beamforms the maximum power, as in Sekihara et al. (2004).

### Temporally adaptive HMM beamforming

In this work we use an approach that augments the HMM inference with an LCMV beamformer, to produce a temporally adaptive beamformer. This works by assuming that the data covariance matrix varies over time. This means that the mean and standard deviations of the source reconstructed time courses now depend on time varying weights and covariance matrices, and are given by:

(14) $$\begin{array}{l} \hat{m}\left(r_{i},t\right)=W\left(r_{i},t\right)y\left(t\right)\\ \mathrm{std}\left(\hat{m}\left(r_{i},t\right)\right)={\left(W\left(r_{i},t\right)C_{y}\left(t\right)W{\left(r_{i},t\right)}^{T}\right)}^{1/2} \end{array}$$

where

(15) $$W\left(r_{i},t\right)={\left(H{\left(r_{i}\right)}^{T}C_{y}{\left(t\right)}^{-1}H\left(r_{i}\right)\right)}^{-1}H{\left(r_{i}\right)}^{T}C_{y}{\left(t\right)}^{-1}$$

where *C_y_*(*t*) is the data covariance matrix at time *t*. We use the state time course from the HMM inference on the same data, *y*, to determine *C_y_*(*t*):

(16) $$C_{y}\left(t\right)=\Sigma_{u\left(t\right)}$$

where

(17) $$\Sigma_{u\left(t\right)}=\Sigma_{\left(u\left(t\right)=k\right)}=\frac{1}{T_{k}-1}\sum_{j=1}^{T_{k}}\left(y\left(t_{k}\left(j\right)\right)-{\bar{y}}_{k}\right){\left(y\left(t_{k}\left(j\right)\right)-{\bar{y}}_{k}\right)}^{T}$$

and where {*t*_*k*_(1) … *t*_*k*_(*T*_*k*_)} is the set of *T_k_* time points for which state *k* is the most probable (as given by Eq. (4)), and y̅*_k_* is the mean over those time points.

### Deriving z-statistical time-courses

We assume that the neural activity at location *r_i_* and time *t*, *m*(*r_i_*, *t*), can be approximated using a Normal distribution:

(18) $$m\left(r_{i},t\right)\sim N\left(\hat{m}\left(r_{i},t\right),\mathrm{std}{\left(\hat{m}\left(r_{i},t\right)\right)}^{2}\right)$$

where $\hat{m}\left(r_{i},t\right)$ and $\mathrm{std}\left(\hat{m}\left(r_{i},t\right)\right)$ are given by Eqs. (11), (13) for the temporally stationary beamformer, and by Eq. (14) for the temporally non-stationary HMM beamformer. A scaled source “*z-statistical*” time course could then be calculated as:

(19) $$z\left(r_{i},t\right)=\hat{m}\left(r_{i},t\right)/\mathrm{std}\left(\hat{m}\left(r_{i},t\right)\right)$$

This represents a time course of z-statistical values, which corrects for the variability of the sensitivity of the sources over space *and* HMM states. The reader should be aware that this is not the same as the pseudo-z-statistic (Vrba and Robinson, 2002) (also known as the neural activity index (Van Veen et al., 1997)), in which the projected data are scaled, not by the best estimate of the standard deviation, but by the estimated standard deviation of the noise (i.e. $\hat{m}\left(r_{i},t\right)/\sigma_{e}{\left[WW^{T}\right]}^{0.5}$, where *σ_e_* corresponds to the estimated noise standard deviation at all of the MEG sensors).

### Deriving z-statistical power time-courses

In the datasets looked at in this paper, it will be *power* (envelope) time courses that we use to compare between task conditions, or as the basis for seed-based correlation. Hence we need to be able to estimate appropriate z-statistical power time courses, which appropriately corrects for the variability of the sensitivity of the sources over space *and* HMM states.

With the temporally stationary beamformer, the standard deviation of the estimate of the neural activity, $\mathrm{std}\left(\hat{m}\left(r_{i},t\right)\right)$, is given in Eq. (13). In this case we could simply compute the z-statistical time courses using Eq. (19) and then perform the power calculation, i.e. the Hilbert transform, to get a z-statistical power time course as:

(20) $$z^{s}\left(r_{i},t\right)=H\left(z\left(r_{i},t\right)\right)$$

where H(x) is the Hilbert envelope amplitude of x.

However, for the time-varying HMM beamformer, the standard deviation, $\mathrm{std}\left(\hat{m}\left(r_{i},t\right)\right)$ is given by Eq. (14). Crucially, this means that the standard deviation now varies over time, since it is computed independently for each state of the HMM model. This means that a more sophisticated approach is needed to calculate the z-statistical power time courses. To do this we use Monte Carlo sampling to allow us to take the probabilistic description of the time courses, given by Eq. (18), and convert them into a probabilistic description (in the form of a Normal distribution) of the corresponding Hilbert envelope time courses.

In practice, we sample a time course from the Normal distribution in Eq. (18). This corresponds to constructing a time course by sampling a value for each time point from the Normal distribution with mean and variances given in Eq. (18). We can then compute the Hilbert envelope for the sampled time course. We repeat this process 100 times to get 100 sampled Hilbert envelope time courses. The standard deviation over samples, *S*(*r_i_*, *t*), is then computed at each time point from these Hilbert envelope time courses, to give an *approximate* Normal distribution on the power at location *r_i_* and time *t*, *μ*(*r_i_*, *t*), as:

(21) $$\mu \left(r_{i},t\right)\sim N\left(H\left(\hat{m}\left(r_{i},t\right)\right),S{\left(r_{i},t\right)}^{2}\right)$$

A scaled source z-statistical power time course could then be calculated as:

(22) $$z^{\mathrm{ns}}\left(r_{i},t\right)=H\left(\hat{m}\left(r_{i},t\right)\right)/S\left(r_{i},t\right)$$

Calculating 100 Monte Carlo simulations for each voxel is time-consuming, and so we also consider using a much more computationally efficient approach where we approximate the standard deviation, *S*(*r_i_*, *t*), of the estimate of the Hilbert envelope time course as being proportional to the standard deviation of the raw time courses, i.e.

(23) $$S\left(r_{i},t\right)\propto \mathrm{std}\left(\hat{m}\left(r_{i},t\right)\right).$$

## Simulated data

### Methods: simulated data

Simulations were undertaken using MEG system geometry based on the third order synthetic gradiometer configuration of a 275 channel whole head CTF MEG system. The location of the brain anatomy with respect to the sensors was taken from a real experimental recording as described for the real MEG resting state data (described later) with a sampling rate of 150 Hz and a length of 500 s. Additive Gaussian noise at three (power) Signal-to-Noise Ratio (SNR) levels (4.001 × 10^− 3^, 1.440 × 10^− 3^, 0.735 × 10^− 3^) were added in sensor space, with different random sampling for each realisation of the simulated data.

A dipolar “signal” source was simulated, located in the left primary motor cortex at the MNI coordinate [41, − 25, 49] mm. This “signal” source time-course comprised Gaussian random noise with unit standard deviation (arbitrary units (AU)), sampled differently for each simulated dataset realisation.

**Fig. 1:**
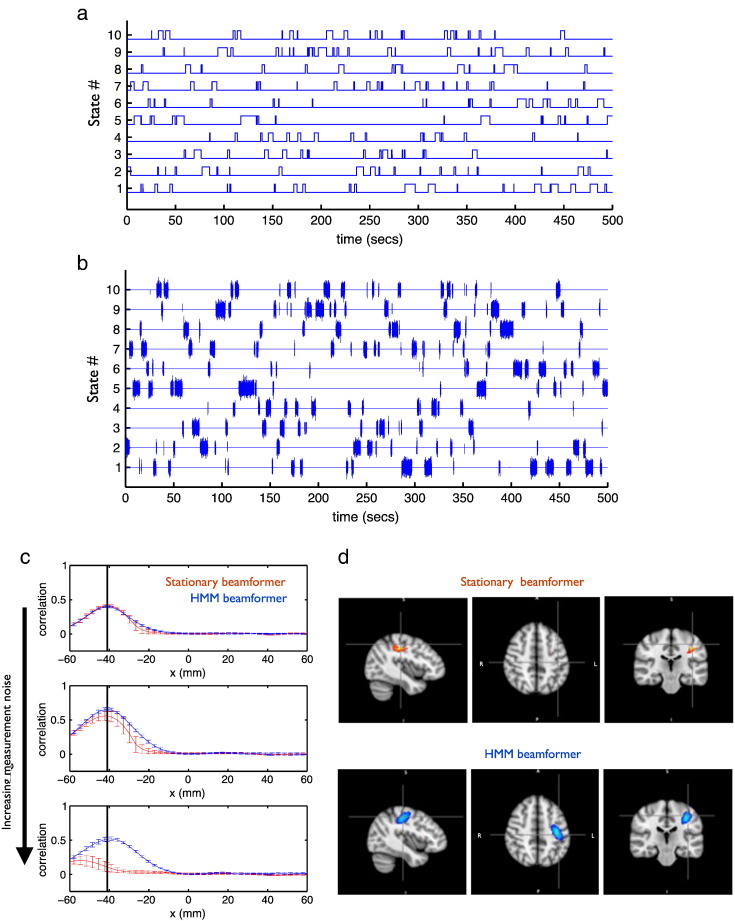
Example of data simulated to contain time-varying “confound” sources using a 10 state HMM. The resulting HMM state time courses are shown in (a), and the resulting simulated signals (in brain space) are shown in (b). These “confound” sources are placed at the MNI coordinates listed in Table 1, along with a unit variance Gaussian random “signal” source (not shown) placed in the left motor cortex (LMC) to create simulated MEG data. Comparisons of standard stationary beamformer (red) and HMM beamformer (blue) using the simulated data. (c) Correlation over time (and over all states) between the beta-band power time courses of the beamformed data and the known signal in the LMC, as x is varied while fixing y = − 25 mm and z = 49 mm with different amounts of sensor measurement noise. Error bars show standard deviation over 10 realisations. (d) T-statistic maps of the correlation with the known beta-band power time course for a single realisation of the simulated data at the highest measurement noise, thresholded at the 99th percentile; the cross hairs are at the true location of the signal.

Ten dipolar temporally non-stationary “confound” sources, located at the MNI coordinates in Table 1, were also simulated using a 10 state HMM with a transition probability of 0.0002 between all states (corresponding to an average state life-time of 2 s). Example state time courses are shown in blue in Fig. 1a. When state k is active, the source time course for dipole k is sampled from Gaussian random noise with standard deviation equal to 10 (AU) (sampled differently for each simulated dataset realisation), and otherwise the source time course has zero standard deviation. An example of the resulting simulated “confound” source signals are shown in Fig. 1b.

**Table 1:** MNI coordinates used for the “confound” sources in the simulated data.

State #	MNI coordinate (mm)
1	50 − 62 26
2	− 50 − 62 26
3	26 32 40
4	− 26 32 40
5	56 − 62 16
6	− 56 − 62 16
7	− 4 50 14
8	30 − 14 − 16
9	− 30 − 14 − 16
10	41 − 25 49

All simulated dipoles are projected into brain space using realistic MEG lead fields to create simulated MEG sensor space data. For either “signal” or “confound” sources, the source orientations were taken as being tangential to the radial orientation but randomised with respect to the azimuthal direction.

These simulated data were then source reconstructed using either a standard stationary beamformer, or the HMM beamformer using a HMM with 10 states. In both cases the beamformer was applied to a reduced subspace with a dimensionality of 100 (Hunt et al., 2012, Woolrich et al., 2011).

Following beamformer projection, the z-statistical power time courses were calculated using Eq. (20) for the standard stationary beamformer, and Eq. (22) for the HMM beamformer, with *S*(*r_i_*, *t*) estimated using Eq. (23). The z-statistical power time courses at each voxel were then low pass filtered with a moving average filter with a width of 0.1 s. The metric of performance used was the correlation (or t-statistic) over all time (and therefore over all states) between the beta-band z-statistical power time courses and the “ground-truth” beta-band power time-course from the left primary motor cortex. The “ground truth” simulated beta band time course was obtained by applying a beta band Hilbert transform on the raw simulated time course placed in the left primary motor cortex.

### Results: simulated data

Fig. 1c shows the comparison of the standard stationary beamformer and HMM beamformer using the simulated data demonstrated in Figs. 1a and b. The plots show the correlation of the beamformed signal with the known signal in the left motor cortex as the x-coordinate is varied, and as the amount of sensor space measurement noise is varied. This is computed for 10 realisations of the simulated data, with the error bars showing the standard deviation. At low sensor noise, both approaches perform well. However, as the noise increases the standard stationary beamformer's erroneous assumption of stationary covariance starts to cause problems and the performance degrades, whereas the performance of the HMM beamformer is maintained. Fig. 1d shows this difference in the performance at the highest noise level for a single realisation of the simulated data using a beamformer grid with 6 mm spacings.

## Resting data

### Methods

#### Data collection

The subject was asked to lie in the scanner and view a centrally presented fixation cross while 300 s of MEG data was recorded. The MEG data were acquired using a 275 channel CTF whole-head system (MISL, Coquitlam, Canada) at a sampling frequency of 600 Hz, and synthetic 3rd order gradiometer correction was applied to reduce external interference. Head localisation within the MEG helmet was achieved using three electromagnetic head position indicator (HPI) coils (placed at three fiducial points: nasion, left and right pre-auricular points). By periodically energising these coils the head position within the MEG sensor array was identified. Prior to data acquisition, the HPI coil locations and the subject's head shape were digitised using a Polhemus Isotrack system. Structural MR images for each subject were acquired using a Philips Achieva 3 T MRI system (MPRAGE; 1 mm isotropic resolution, 256 × 256 × 160 matrix, TR = 8.1 ms, TE = 3.7 ms, TI = 960 ms, shot interval = 3 s, flip angle = 8° and SENSE factor 2). The locations of the MEG sensors were co-registered to the brain anatomy by matching the digitised head surface to the head surface extracted from the anatomical image.

#### Data analysis

Periods of data containing large artefacts were identified by visual inspection, and discarded. The registration of the subject's head shape to their structural MRI was carried out using SPM8 (www.fil.ion.ucl.ac.uk/spm). MEG data were then frequency filtered into the 13 to 30 Hz (β) band and projected into source space using either the standard stationary or HMM beamformers. In both cases the beamformer was applied to a PCA reduced subspace with a dimensionality of 100. Voxels were placed on a regular 5 mm grid spanning a grey matter mask across the entire brain. Following beamformer projection, the z-statistical power time courses were calculated using Eq. (20) for the standard stationary beamformer, and Eq. (22) for the HMM beamformer. The z-statistical power time courses at each voxel were then low pass filtered with a moving average filter with a width of 0.5 s, and downsampled to 2 Hz, based on findings from a previous study optimising the moving average filter width (Luckhoo et al., 2012).

#### Seed-based correlation and spatial leakage reduction

A seed location was defined in the right motor cortex at the MNI coordinate [42 − 26 48] mm. Seed-based regression t-statistic maps were produced by regressing the seed voxel's low pass filtered and down-sampled z-statistical power time course onto all other test voxel low pass filtered and down-sampled z-statistical power time courses.

Note that prior to the Hilbert envelope calculations, we reduced the effect of zero-time-lag spatial leakage between the seed and test voxels using the approach described in Brookes et al. (2012) and Hipp et al. (2012). This uses linear regression and subtraction of the raw time series at the seed location from the raw time series at each of the test voxels, and has been shown to significantly reduce leakage and enhance connectivity estimates. This was carried out independently for each HMM state.

### Results — resting data

**Fig. 2:**
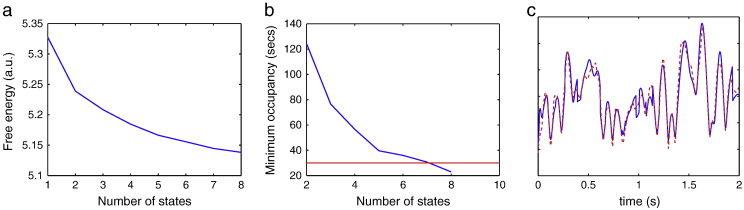
Resting state MEG dataset. Plots showing the effect of varying the assumed number of states in the HMM: (a) on an approximation to the model evidence (i.e. the free energy), and (b) on the occupancy (i.e. amount of time spent in a state) for the state that has the minimum occupancy. The red line indicates the smallest minimum occupancy (30 s) that is considered acceptable to give a good estimate of the covariance matrix for use in beamforming. (c) Plot comparing the z-statistical power time courses for the seed voxel; where the standard deviations of the estimate of the power time course are estimated either using Monte Carlo simulations (red), or by assuming that it is proportional to the raw time course standard deviation (blue).

Fig. 2 shows the effect of varying the assumed number of states in the HMM for the resting state MEG dataset. The model evidence (estimated via the negative of the free energy) monotonically increases up to a model order of 8, suggesting that the Bayes-optimal model may require an even higher number of states. However, as shown in Fig. 2b, the occupancy (i.e. amount of time spent in a state) for the state that has the minimum occupancy drops below 30 s with a model order of 8. This is a more meaningful metric with regard to the intended use of the HMM, specifically the estimation of data covariance matrices for use in the standard stationary beamformer. As shown in Brookes et al. (2008) and Woolrich et al. (2011), the amount of data required to give a good estimate of the covariance matrix for data with bandwidth of the beta band (13 to 30 Hz) is of the order of about 30 s. As a result of this we use an HMM with 7 states for the rest of the analyses in this paper.

**Fig. 3:**
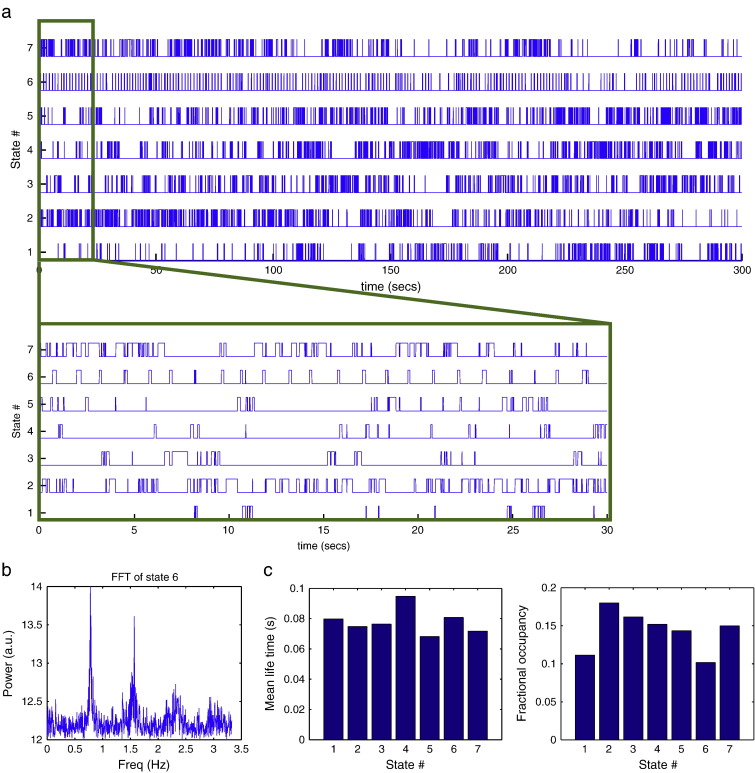
Results of fitting the HMM model with 7 states to the resting state MEG dataset. (a) Inferred HMM state time courses, with the first 30 s also shown zoomed in. The 6th state can be seen to be visited regularly about every 1 s, which is confirmed by the FFT of its state time course in (b), consistent with this state representing a cardiac related signal. (c) Mean life time and fractional occupancy of the states are also shown.

**Fig. 4:**
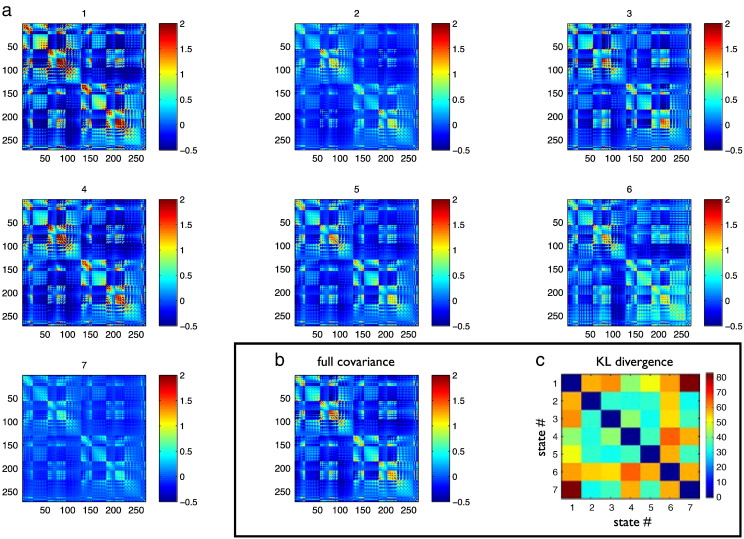
(a) Data covariance matrices computed by pooling the data during the points in time when those states are active (as given by the state time courses in Fig. 3a). (b) The data covariance matrix of the full data. (c) The symmetric KL computed between all seven state covariance matrices. Results are shown for the resting state MEG dataset.

Fig. 3 shows the result of fitting the HMM model with 7 states to the resting state MEG dataset, including the inferred HMM state time courses. In particular, state 6 is visited regularly about every 1 s, which is confirmed by the FFT of its state time course in Fig. 4b. This is consistent with state 6 corresponding to a cardiac related artefact.

The data covariance matrices are computed by pooling the data during the points in time when the states are active (as given by the state time courses in Fig. 4a), and are shown in Fig. 4. The differences between the state covariance matrices are quantified using the Kullback–Leibler (KL) divergence, as shown in Fig. 4c.

Fig. 2c compares the z-statistical power time courses for the seed voxel, where the standard deviation of the power time course is estimated either using Monte Carlo simulations (Eq. (22)), or by assuming that it is proportional to the raw time course standard deviation (Eq. (23)). This demonstrates the similarity of the z-statistical power time courses obtained using either approach. Hence, for computational efficiency, we estimate the standard deviation of the power time course by assuming that it is proportional to the raw time course standard deviation (Eq. (23)), in the rest of the analyses in this paper.

**Fig. 5:**
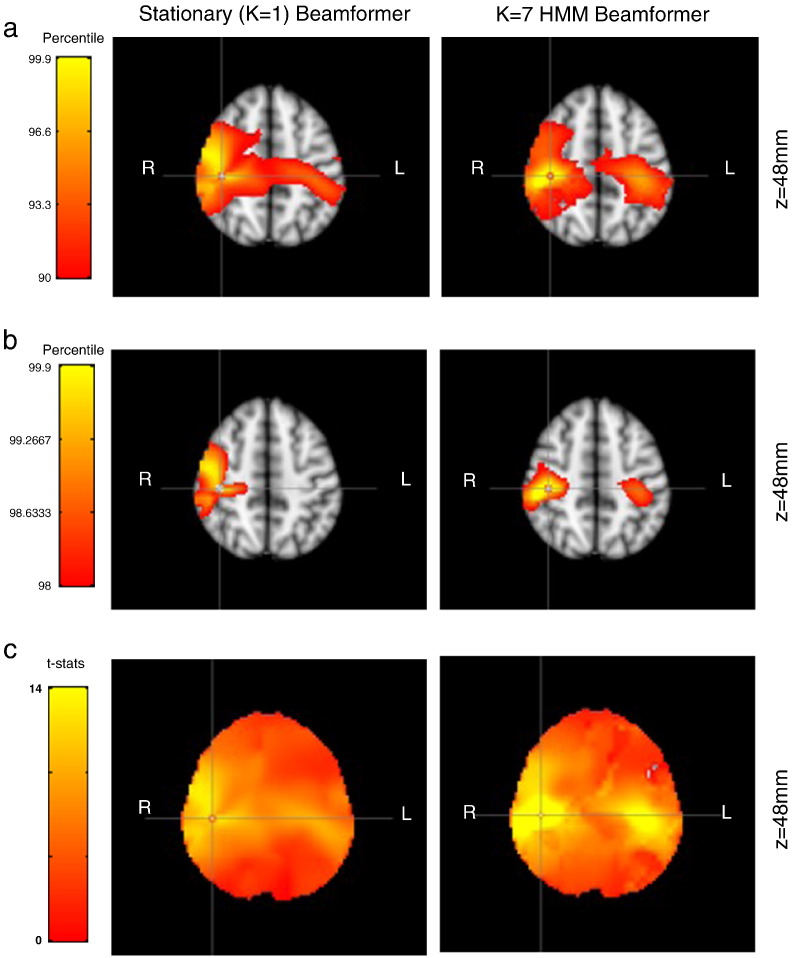
Comparison of standard stationary beamformer [left] and a 7 state HMM beamformer [right], for the resting state MEG dataset. Images show t-statistics of a seed-based regression of the low-pass filtered beta band power, thresholded at (a) the 90th percentile, and (b) the 98th percentile. Cross hairs are located at the seed voxel location [42 − 26 48]. (c) The zero meaned beta-power z-statistical power time courses used in the seed-based correlation calculations, for the seed voxel (red) and in the left motor cortex [− 42 − 26 48] (blue).

Fig. 5b shows spatial maps of t-statistics corresponding to the correlation of beta band power with the seed voxel in the right motor cortex (thresholded at the 98th percentile of each approach). At this threshold the standard stationary beamformer only shows correlation around the seed. Whereas the HMM beamformer shows bilateral correlation with the seed voxel; this is consistent with left–right motor cortex power–power coupling demonstrated in previous papers (Brookes et al., 2011; Brookes et al., 2011; Hipp et al., 2012). Fig. 5a shows the same images, but with a lower threshold at the 90th percentile of each approach. The standard stationary beamformer now shows bilateral motor areas; however, it is not as lateralised as the HMM beamformer approach.

## Motor task

### Methods

The motor task MEG data was acquired in the same manner as the resting state data, as described in the section “Resting Data”. The subject was a right-handed female who undertook a motor task (see Brookes et al., 2011 for details), which included 30 s blocks of rest, left finger tapping, right finger tapping, or simultaneous left + right finger tapping. Here we look for beta band power changes between simultaneous left + right finger tapping and rest.

The data analysis is the same as for the resting data. As with the resting state data the z-statistical power time courses were calculated using Eq. (20) for the standard stationary beamformer, and Eq. (22) for the HMM beamformer. The z-statistical power time courses at each voxel were then low pass filtered with a moving average filter with a width of 0.1 s, and downsampled to 10 Hz.

However, rather than then doing seed-based correlation, we fit a multiple regression (or General Linear Model (GLM)) to the z-statistical power time courses using a design matrix containing 4 regressors to model the different conditions (rest, left finger tapping, right finger tapping, simultaneous left + right finger tapping). We then contrasted the simultaneous left + right finger tapping regression parameter with respect to the rest regression parameter, and computed uncorrected t-statistics using standard GLM statistics (Luckhoo et al., 2012).

### Results — motor task

**Fig. 6:**
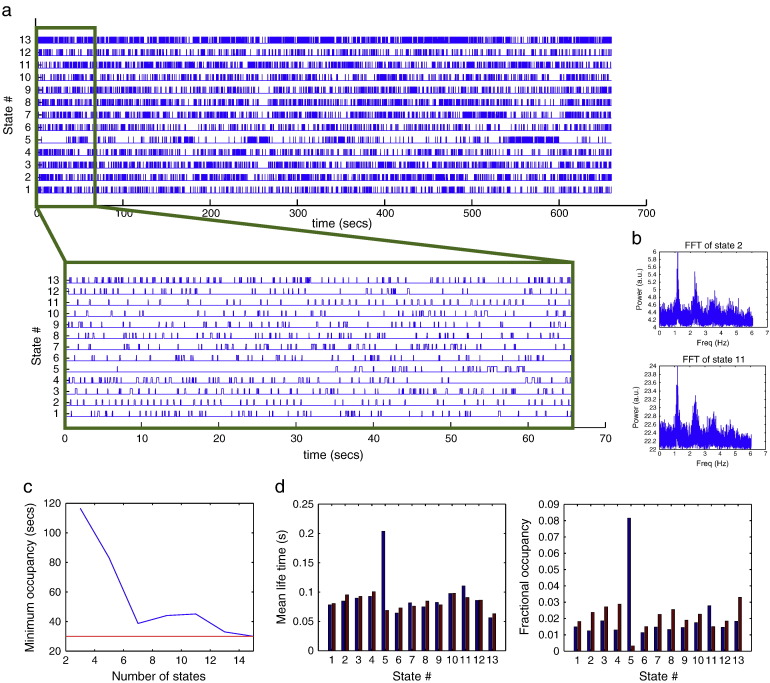
Results of fitting the HMM model with 13 states to the real motor (finger tapping) MEG dataset. (a) Inferred HMM state time courses, with the first 65 s also shown zoomed in. States 2 and 11 can be seen to be visited regularly about every 1 s, which is confirmed by the FFT of their state time course in (b), consistent with this state representing a cardiac related signal. (c) Effect of varying the assumed number of states in the HMM on the occupancy (i.e. amount of time spent in a state) for the state that has the minimum occupancy. (d) Mean life time and fractional occupancy of the state time courses within rest (blue) and finger tapping (red) epochs.

Fig. 6c shows that the occupancy (i.e. amount of time spent in a state) for the state that has the minimum occupancy, drops below 30 s with a model order of 14. As a result we use an HMM with 13 states for the rest of the analysis. This is considerably higher than the number of states for the resting state analysis (7). However, this is primarily due to the motor task data being approximately twice as long.

Fig. 6a shows the result of fitting the HMM model with 13 states to the motor task MEG dataset, including the inferred HMM state time courses. There are two different states that appear to correspond to the cardiac cycle, i.e. states 2 and 10, as confirmed by the FFT of their state time course in Fig. 6b. Fig. 6d shows the mean life-time and fractional occupancy of the state time courses. As with the resting dataset the states are short lived, with mean life times on the order of 100 ms. Fig. 6d also shows the mean life-time and fractional occupancy broken down for the two conditions of rest and finger tapping. State 5 shows a particularly strong difference between conditions, appearing mostly in the rest condition, with a longer mean life-time than the other states.

**Fig. 7:**
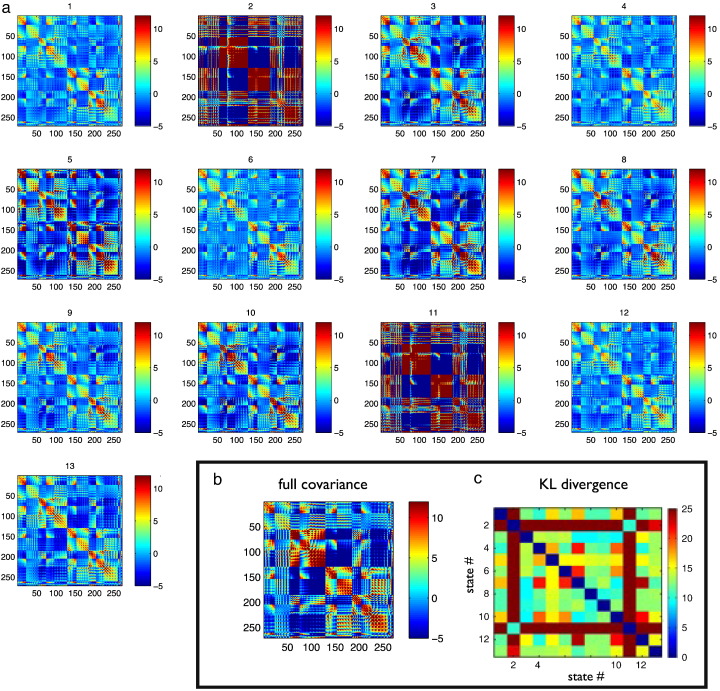
(a) Data covariance matrices computed by pooling the data during the points in time when those states are active (as given by the state time courses in Fig. 6). (b) The data covariance matrix of the full data. (c) The symmetric KL computed between all seven state covariance matrices. Results are shown for the real motor (finger tapping) MEG dataset.

The data covariance matrices are computed by pooling the data during the points in time when those states are active (as given by the state time courses in Fig. 6a), and are shown in Fig. 7. Fig. 9, Fig. 10 show a comparison of the t-statistic spatial maps from the standard stationary beamformer and the 13 state HMM beamformer. The t-statistics are calculated from the multiple regression (GLM) and show decreases in beta-band power between simultaneous left + right finger tapping and rest. Qualitatively, the activity has increased spatial specificity in the HMM beamformer compared with the standard stationary beamformer. For example, the standard stationary beamformer tends to have just one extended cluster of activation in each hemisphere centred on the primary somato-sensory cortex. In contrast, at the same percentile threshold, the HMM beamformer is able to find multiple, distinct clusters of activity corresponding to plausible areas of the motor network.

**Fig. 8:**
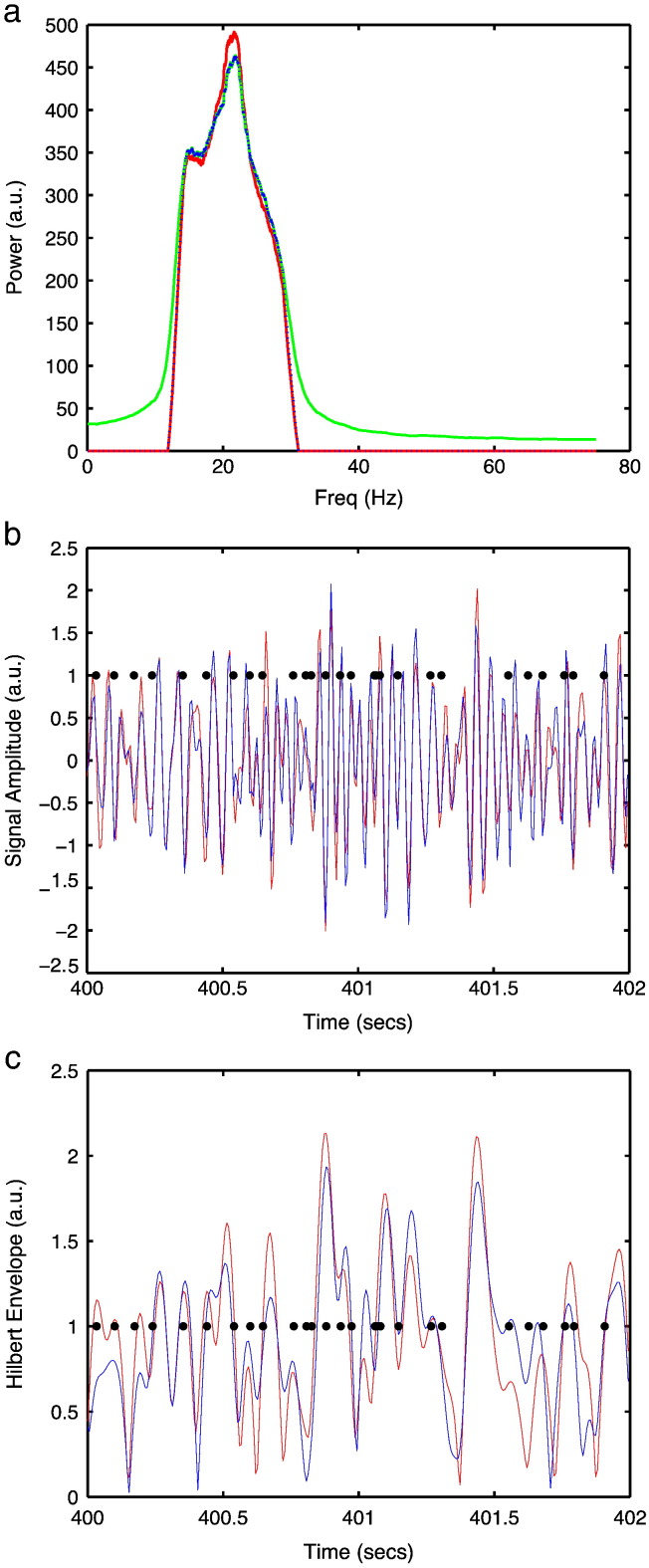
(a). Power spectral density of raw source reconstructed time course for the HMM beamformer (green), stationary beamformer (red), and HMM beamformer followed by beta band bandpass filtering (black). Representative 2 s time segment of the raw source reconstructed time course (b), and the corresponding Hilbert envelope time course (c), for the stationary beamformer (red) and the HMM beamformer (blue), where the black dots indicate when there is a switch in HMM state. Note that there is no qualitative evidence of strong discontinuities or oscillatory components due to the fast HMM state switching. Results are shown for the left motor cortex (MNI coords: [36 − 18 52] mm) in the finger tapping MEG dataset.

**Fig. 9:**
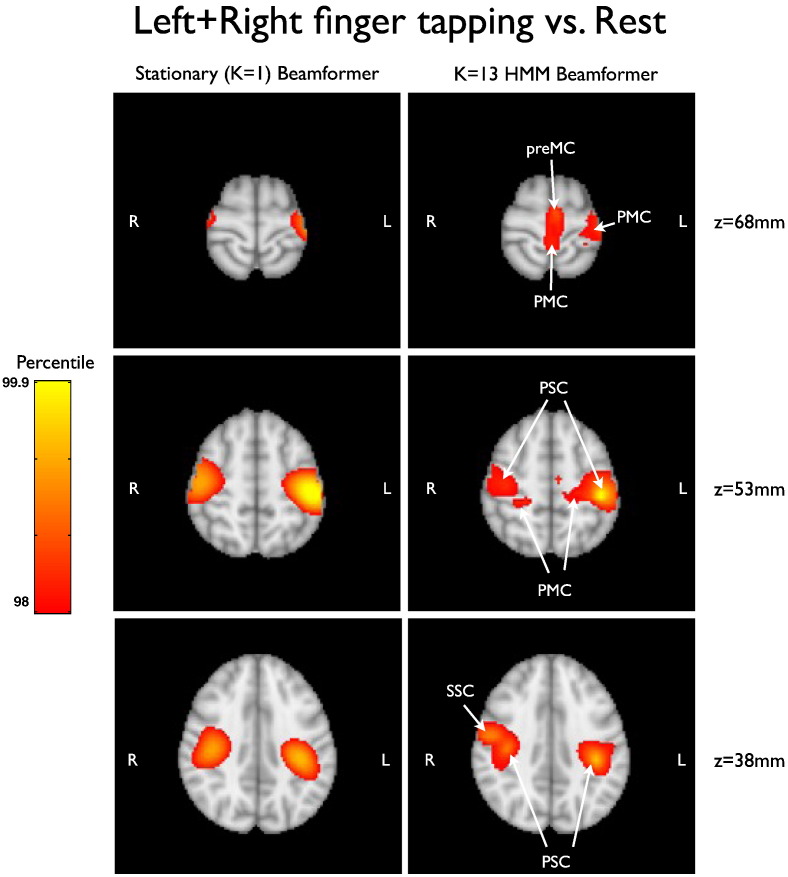
Comparison of standard stationary beamformer [left] and a 13 state HMM beamformer [right], for the finger-tapping MEG dataset. Images show three slices of the uncorrected t-statistic spatial maps, indicating beta band power decreases for simultaneous left + right finger tapping versus rest, thresholded at the 98th percentile. [PMC — primary motor cortex, PSC — primary somato-sensory cortex, preMC — premotor cortex (supplementary motor area), SSC — secondary somato-sensory cortex.]

**Fig. 10:**
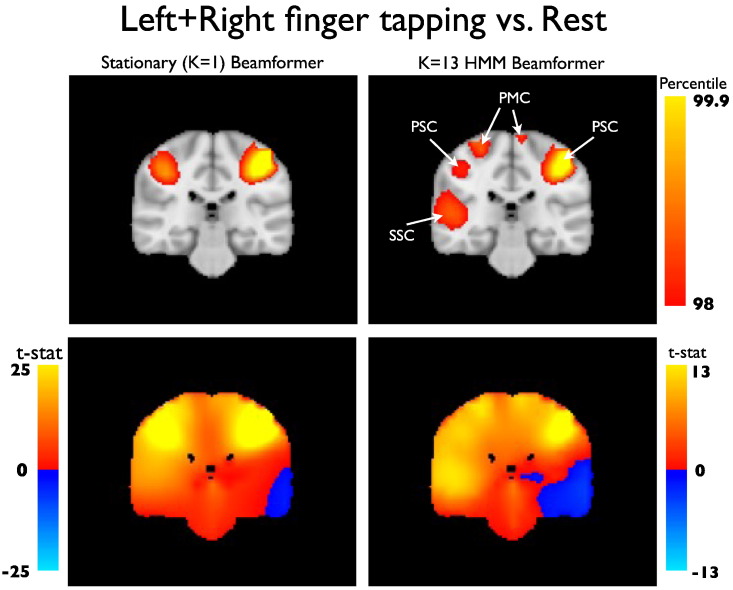
As in Fig. 9 but with images showing the y = − 28 mm slice of the uncorrected t-statistic spatial maps, indicating beta band power decreases for simultaneous left + right finger tapping versus rest, [top] thresholded at the 98th percentile, and [bottom] unthresholded.

#### Comparison with ICA denoising

In Fig. 6 we saw that two states in particular are likely associated with the cardiac cycle. It is possible that the qualitative improvements attributed to the HMM beamforming could be solely due to its ability to identify (and downweight) artefacts such as this. In this case, the approach might be expected to perform similar to using ICA denoising in sensor space, followed by standard stationary beamforming. To investigate this possibility we carried out standard stationary and HMM beamformer analyses, with and without ICA denoising. ICA was carried out using fastICA via the symmetric approach and with a dimensionality of 60 (Hyvärinen, 1999). Two components were manually identified as corresponding to cardiac artefact, and two components as eye/eye-blink movement related artefact. These components were then subtracted from the data (Mantini et al., 2011), with the same operation applied to the lead fields.

**Fig. 11:**
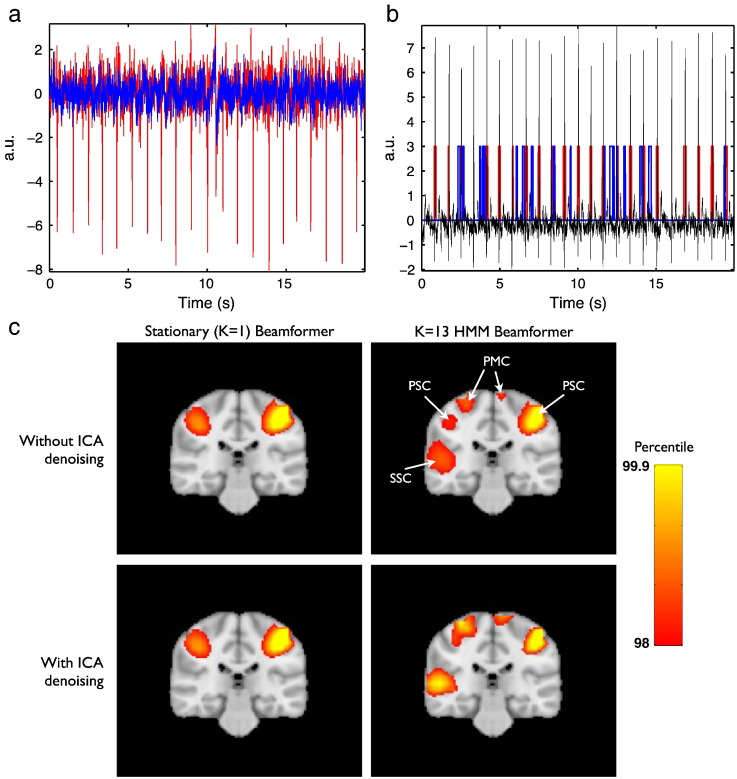
Effects of ICA denoising. (a) First 20 s of sensor time course for the sensor showing the highest correlation with the main ECG IC time course, before (red) [correlation = − 0.82] and after (blue) [correlation = − 0.03] removal of the IC component corresponding to the ECG artefact. (b) First 20 s of HMM state time courses for the HMM state with the highest correlation with the main ECG IC time course when running the HMM inference on data, before (red) [correlation = 0.23] and after (blue) [correlation = 0.02] removal of the IC component corresponding to the ECG artefact. The IC time course identified as the ECG artefact is shown for comparison (black). (c) Comparison of standard stationary beamformer [left] and a 13 state HMM beamformer [right], for the finger-tapping MEG dataset, shown with [bottom] and without [top] ICA denoising having been applied in sensor space. Images show the z = − 28 mm slice of the uncorrected t-statistic spatial maps.

Fig. 11a shows the first 20 s of the sensor time course for the sensor showing the highest correlation with one of the IC time courses identified as being ECG related, before [correlation = − 0.82] and after [correlation = − 0.03] removal of the IC component corresponding to the ECG artefact for the finger tapping data. Standard stationary and HMM beamforming was then applied. Fig. 11b shows the first 20 s of HMM state time courses for the HMM state with the highest correlation with the main ECG IC time course when running the HMM inference on data, before [correlation = 0.23] and after [correlation = 0.02] removal of the IC component corresponding to the ECG artefact. Fig. 11c shows a comparison of the standard stationary beamformer and the 13 state HMM beamformer for finger-tapping versus rest with and without ICA denoising having been applied in sensor space. This suggests that the HMM beamformer is doing more than ECG/eye movement denoising alone.

#### Comparison with a sliding window approach

**Fig. 12:**
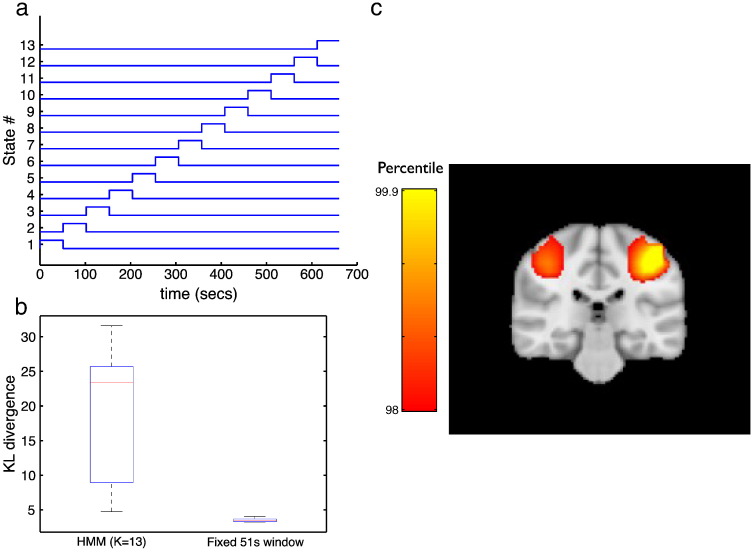
Fixed sliding time window approach (with window width 51 s). This is equivalent to the HMM approach but where the HMM state time courses are fixed as shown in (a) (rather than inferred from the data). (b) Shown is the distribution of the KL divergences between the full stationary data covariance matrix and the data covariance matrices estimated using (left) the HMM state time courses for the K = 13 HMM approach, and (right) the fixed sliding time courses shown in (a). (c) Shown is the result of using the fixed sliding time window beamformer approach for comparison with the maps in Fig. 10.

It would be expected that a sliding window approach (similar to that used in Dalal et al., 2008) with time windows of several seconds would be blind to the short-lived states being found by the HMM inference (~ 100 ms). To confirm this we carried out a sliding window analysis on the finger tapping data. The results are shown in Fig. 12. This used fixed time windows with widths of 50 s (matched to have the same average occupancy as the HMM state time courses). Note that this is equivalent to the HMM approach but where the HMM state time courses are fixed as shown in Fig. 12a. Fig. 12b shows the distribution of the KL divergences between the full stationary data covariance matrix and the data covariance matrices estimated using the HMM state time courses or the fixed sliding window time courses. This indicates a much lower KL divergence (with respect to the global stationary covariance) for the sliding window covariances than for the HMM-derived covariances. Fig. 12c shows the result of using the fixed sliding time window beamformer approach. This looks very similar to the stationary beamformer spatial maps in Fig. 10.

#### State discontinuities

In the HMM beamformer the raw time courses are obtained from beamformer weights computed for different HMM states, where consecutive time points may be derived from different states. This has the potential to introduce discontinuities on average every 100 ms in the source time courses; and could introduce a ~ 10 Hz artefact that could have an adverse effect on the computed shape of the Hilbert envelopes. Fig. 8a shows the power spectral density of raw source reconstructed time course for the HMM beamformer compared with the standard stationary beamformer, before and after beta band bandpass filtering, in the left motor cortex (MNI coords: [36 − 18 52] mm) in the finger tapping MEG dataset. There is no qualitative evidence of a ~ 10 Hz component. Figs. 8b and c shows a representative 2 s time segment of the raw source reconstructed time course, and the corresponding Hilbert envelope time course, alongside when there is a switch in HMM state. There is no qualitative evidence of strong discontinuities due to the fast HMM state switching.

#### Within-state analysis

**Fig. 13:**
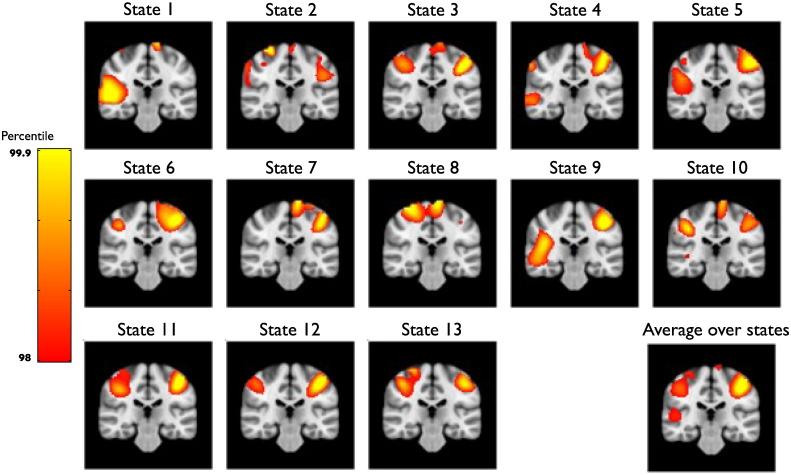
Result of running the GLM separately within each HMM state. T-statistic maps show comparison of beta power in simultaneous left + right finger tapping versus rest separately for all 13 HMM states in the finger-tapping MEG dataset, and [bottom-right] the result of doing a fixed-effects averaging over all of these 13 analyses. Images are thresholded at the 98th percentile.

The HMM beamformer approach presented so far reconstructs beta power time courses, upon which a single GLM has been run to compare beta power in simultaneous left + right finger tapping versus rest (e.g. Fig. 10). However, an alternative strategy is to run separate GLMs on each HMM state. Fig. 13 shows the results of doing this within-state GLM analysis. This has the advantage in that it can alleviate the potential impact of any discontinuities on the GLM due to fast HMM state transitions, and also allows us to see the underlying effects within each state. Fig. 13 shows that each individual state produces quite different spatial maps, with different subsets of the motor network being recruited. Fig. 13 shows the net result of these constituent parts via a fixed-effects averaging (Woolrich et al., 2004) over the 13 states. This spatial map looks very similar to the spatial map obtained from the original single GLM analysis shown in Fig. 10.

## Discussion

We have presented a new adaptive time-varying approach to source reconstruction, underpinned by a Hidden Markov Model (HMM). The HMM infers when in time particular states occur, allowing intelligent pooling of data over distinct and potentially short-lived periods in time. This is used to compute time-varying data covariance matrices for use in beamforming, resulting in a source reconstruction approach that can tune its spatial filtering properties to that which is required at different points in time.

While we do not have any absolute ground truth, the results indicate a qualitative improvement in the spatial maps on the resting and motor MEG datasets when using the HMM beamformer compared with the standard stationary beamformer. In the resting data the t-statistics are comparable in the left and the right motor cortex (see Fig. 5). However, in medial cortex the values are reduced in the HMM beamformer, suggesting a slight reduction in the spatial leakage. Investigating the broader effects of non-stationary methods on functional connectivity, including the important issue of controlling for false-positive connections, is an important area for further work.

In the motor dataset the t-statistics in the primary somato-sensory cortex appear reduced, but there appears to be greater spatial specificity with distinct clusters of activation apparent in plausible areas of the motor network including primary motor cortex, primary somato-sensory cortex, premotor cortex, and secondary somato-sensory cortex (see Fig. 9, Fig. 10). This potential improvement cannot be attributed solely to artefact denoising (Fig. 11). A within-HMM state analysis (Fig. 13) suggests that this is being driven by each state exhibiting different subsets of the motor network that have beta power changes between finger tapping and rest.

Most M/EEG source localisation algorithms are based on the assumption of temporal stationarity. The unsupervised classification of the data into stationary blocks using the HMM, will have benefits for any temporally stationary source localisation approach, simply because the within-state sensor data will be more temporally stationary than the sensor data as a whole. Similar advantages to those demonstrated in this paper are likely to be achieved by pre-calculating the HMM state time courses for use in other source localisation approaches, such as multiple sparse priors (Friston et al., 2008).

### State interpretation

In this work, the focus is on finding states of temporally stationary covariance structure, and seeing how we can use this to improve source reconstruction methods such as beamforming. For this objective, there is no need to be able to identify the underlying physical processes that are causing the different HMM states, i.e. we do not need to be able to interpret them as being due to specific artefacts, or due to specific neuronal processes. That said, the HMM inference finds states that live for time periods on the scale of about 100 ms. Intriguingly, this is on the same timescale as EEG microstates. EEG microstates are defined as short periods (~ 100 ms) during which the sensor space topography remains approximately stable. These microstates are thought to correspond to transient coherent activation within resting state networks (Britz et al., 2010, Koenig et al., 2005, Van de Ville et al., 2010). In future work, we will look to see how HMM states found in MEG may relate to EEG microstates, and in turn to resting state networks found using techniques like ICA in resting (Brookes et al., 2011) or task (Luckhoo et al., 2012) MEG data.

### Sliding window approaches

Previous work has looked to estimate covariance matrices at each point in time using sliding time windows (Dalal et al., 2008). However, a sliding window approach is limited to finding transient activity with life times long enough to allow the covariance to be estimated reliably (i.e. using time windows on the order of several seconds). In contrast, the Hidden Markov Model simultaneously infers the covariance matrices for each state *and* the probability of being in a particular state at each point in time. This allows the covariance matrix for a state to be estimated by pooling over all points in time when that state is active, even when each visit to that state may be short-lived. Indeed, we found that the HMM states had average life times on the order of hundreds of milliseconds; and as demonstrated in Fig. 12, a sliding window approach with time windows of several seconds is effectively blind to these short timescales.

### Model order

HMM inference needs an a-priori specification of the number of states. Previous work has explored the performance of the HMM inference with regard to this choice of model order (Rezek and Roberts, 2005). However, in the specific context of using HMMs in beamforming, we intentionally choose a model order that is less than the “correct” model order (see Fig. 2). This is due to the overriding requirement that we want HMM states with enough occupancy to give good estimates of the covariance matrices for the purposes of beamforming.

### State transitions

In this paper we have used a hard transition between states. That is, the time series from the estimated sources have discontinuities at the state boundaries when the weights change. We found no evidence of discontinuities in the reconstructed time courses caused by the rapid switching of the HMM states (see Fig. 8). Nevertheless, an alternative scheme that would alleviate this potential issue would be to make use of the full probabilistic inference on *s_t_*, by using *P*(*s_t_*|*y*) to perform weighted averaging to construct a distinct covariance matrix at every time point. However, this would require computation of unique beamformer weights at every time point (rather than for every state as is possible with the hard transition approach); this would be computationally intensive. A computationally efficient but approximate alternative may be to perform the weighted averaging on the state specific weights instead.

The ability to have different representations of the data at different time points has the potential to increase the spatial information that can be extracted from MEG data. With a temporally stationary approach, the CTF data used in this paper can have at most 275 (the number sensors) independent pieces of information about the spatial activity in brain space. In reality the independent information is likely to be a lot less than this (note that the Elekta Neuromag Signal Source Separation (SSS) Maxfilter approach produces data with a dimensionality of only about 64 (Woolrich et al., 2011)). While the dimensionality cannot be higher than the number of independent sensors at any one time point, a temporally non-stationary approach allows us to span a different subspace at different points in time. This effectively increases the amount of spatial information that can be extracted.
